# Evidence of a link between taboos and sacrifices and resource scarcity of ritual plants

**DOI:** 10.1186/1746-4269-11-5

**Published:** 2015-01-08

**Authors:** Diana Quiroz, Tinde van Andel

**Affiliations:** Naturalis Biodiversity Center, P.O. Box 9517, 2300 RA Leiden, The Netherlands; Wageningen University, Biosystematics, P.O. Box 647, 6700AP Wageningen, The Netherlands

**Keywords:** Africa, Benin, Bwiti, Ethnobotany, Gabon, IUCN Red List, Plant conservation, Threatened species, Vodoun

## Abstract

**Background:**

One of the main obstacles for the mainstreaming of religious traditions as tools for the conservation of nature is the limited applicability of research results in this field. We documented two different restrictions implemented by local people (taboos and sacrifices) related to the use of ritual plants in Benin (West Africa) and Gabon (Central Africa).

**Methods:**

To see whether these restrictions reflected plant scarcity from an etic perspective (official threat status) and an emic viewpoint (perceived scarcity by local people), we conducted 102 interviews with traditional healers and adepts of traditional faiths.

**Results:**

We documented a total of 618 ritual plants, from which 52 species were used in both countries. In Benin, the use of 63 of the 414 ritual plant species was restricted; while in Gabon 23 of the 256 ritual plants were associated with taboos and sacrifices. In Benin, restricted plants were significantly more often officially threatened, perceived as scarce, and actively protected than non-restricted plants. In the more forested and less densely populated Gabon, plants that were perceived as scarce were more often associated to local restrictions than officially threatened species.

**Conclusions:**

These results prove the presence of a form of adaptive management where restrictions are related to resource scarcity and protection of ritual plant species. By providing baseline data on possibly endangered species, we demonstrate how plant use in the context of religious traditions can yield important information for conservation planning.

**Electronic supplementary material:**

The online version of this article (doi:10.1186/1746-4269-11-5) contains supplementary material, which is available to authorized users.

## Background

One of the main obstacles in the mainstreaming of practices associated with religious traditions as tools for the conservation of biodiversity is the insufficient applicability of research results in this field. Over the last two decades, a considerable body of evidence linking religious traditions to nature conservation has been produced in academia [1–6]. However, at the policy level, the integration of these data into conservation planning and implementation remains rather limited, like for example, the almost exclusive attention that sacred natural sites have received as de-facto protected areas. Although this is a laudable achievement, cultural values (which include religious traditions) encompass a wider range of social mechanisms that pose an untapped potential in conservation [7], as they are a form of adaptive management of natural resources based on traditional ecological knowledge [8]. Our work is concerned with practices related to religious traditions that regulate the use of ritual plants, namely, taboos and sacrifices.

Following Evans-Pritchard [9], we consider sacrifices as a symbolic payment prior to the use of a certain plant. Sacrifices have received relatively little attention in circles other than archaeology and social anthropology. Taboos, which we define according to Meyer-Rochow [10] as the prohibition to interact with a plant, are, on the other hand, a topic of interest in several disciplines. In fact, there is a long-standing dissension about their necessity and origin in human societies. Taboos have been found to exist as a means of avoidance of potential health hazards [10–13], or associated with signs of spiritual purity [14]. They have also been viewed as mechanisms for the partitioning of resources, either with the purpose of monopolizing [15] or conserving them [10, 16–18]. While these views might support or refute one another, the discussion has predominantly revolved around food taboos and, particularly, the consumption of wild-caught fish and birds or bush meat [11, 19, 20]. Moreover, only a few of these studies are supported by quantitative analysis based on interviews with local informants [12].

We focused on adepts of different traditional faiths and traditional health practitioners (henceforth healers) in Benin (West Africa) and Gabon (Central Africa) in order to identify ritual plant species. We define ritual plants as those plants that are employed in the context of religious traditions in Benin and Gabon. For example, medicinal plants that are used in religious healing ceremonies, or as amulets, charms, food offerings for spirits or ancestors, are all examples of ritual plants. Likewise, we use the term "non-ritual plants" as those plants that are not used in a religious context. In the same line of argument, we define rituals as ceremonial activities that take place in the context of these traditional religions. In Benin, traditional faiths are officially recognized as state religions and their practice is widely accepted [21]. Situated in the Dahomey Gap, the dry corridor between the Upper and Lower Guinean rain forest blocks, Benin’s vegetation cover comprises, for its greatest part, a forest-savanna mosaic that includes some 2800 plant species [22]. With an estimated flora of over 4700 species [23], Gabon is located in the Guineo-Congolian phytogeographical region [24] and is mainly covered with lowland rain and swamp forests. Although non-traditional religions (e.g. Christianity and Islam) are more widespread than in Benin, traditional religions (or a syncretic version of them) remain important in Gabon.

Our work consisted in documenting taboos and sacrifices associated with the use of plant species in the context of the traditional religious practices of the two countries. Hereinafter, we use the term restrictions to refer to taboos and sacrifices. Our objective was to see whether these restrictions reflected plant scarcity from an etic perspective (official threat status, e.g. IUCN Red List) and an emic viewpoint (perceived scarcity by local people). We expected that the number of officially threatened species would be higher among species associated with taboos or sacrifices (restricted plant species, hereafter) than among those species that were not associated with taboos or sacrifices (non-restricted species, henceforth). Likewise, we expected the proportion of plants perceived as scarce by our informants to be higher among the restricted plants.

Another theoretical assumption relevant for our study stemmed from previous work on ecological niche theory and (food) taboos by Rea and Begossi [12, 25]. These authors departed from an application of the Huntchinsonian niche theory proposed by Hardesty [26]. In human ecology, the niche is elucidated by the amount of variety present and the number of resources used for subsistence by human populations. Rea and Begossi (l.c.) posited that "taboos are a luxury" and that people whose niche comprises a high availability of resources implement them. Following this assumption, we would expect to find a larger number of restricted plant species in Gabon, a country with extensive forest cover and low population density, than in the densely populated Benin which has lost the majority of its forest cover in the last two decades [27].

## Methods

**Figure 1:**
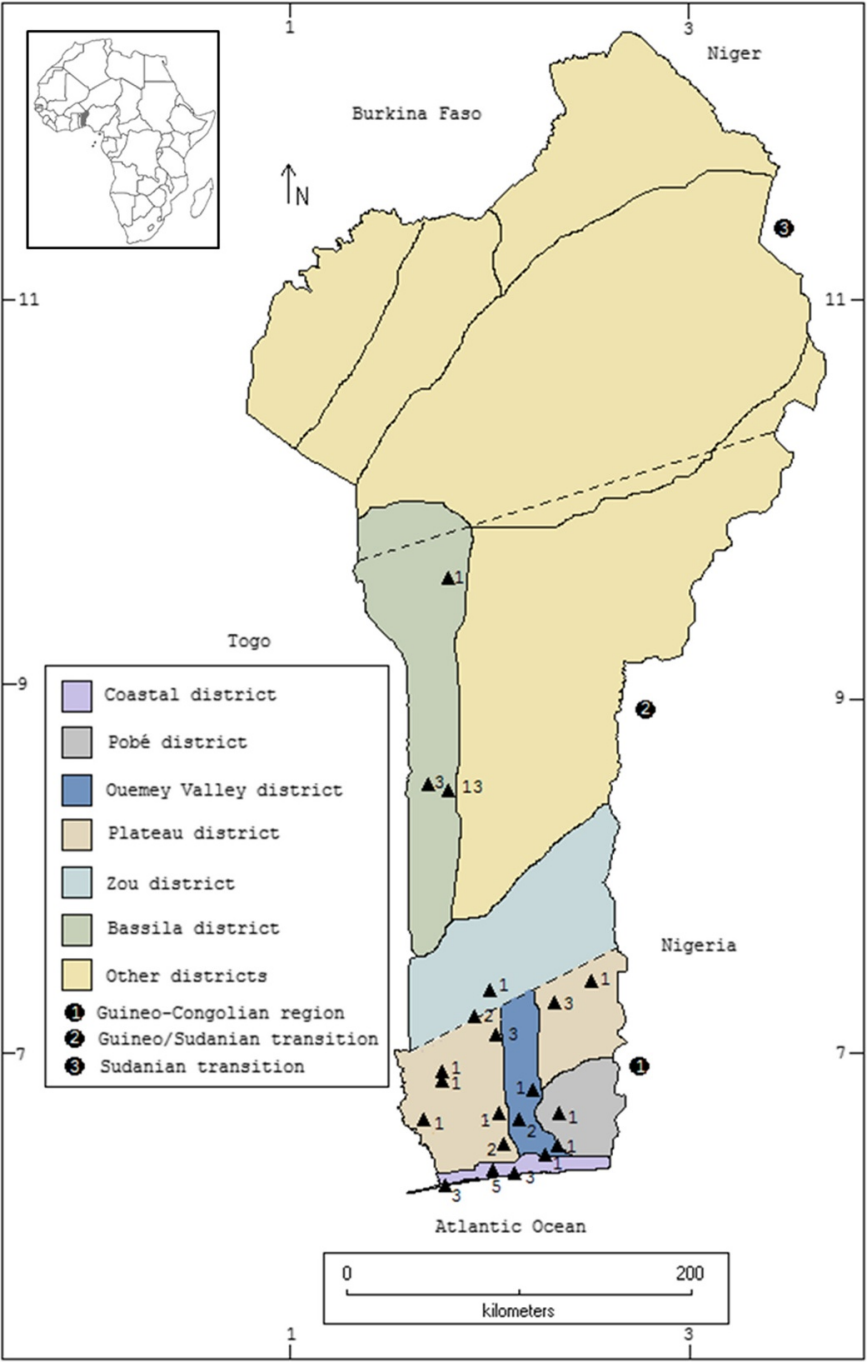
**Map of Benin displaying the different phytogeographical districts and vegetation zones based on plant species composition.** Adapted from Adomou [21]. Triangles indicate surveyed locations. Numbers indicate informants per location.

**Figure 2:**
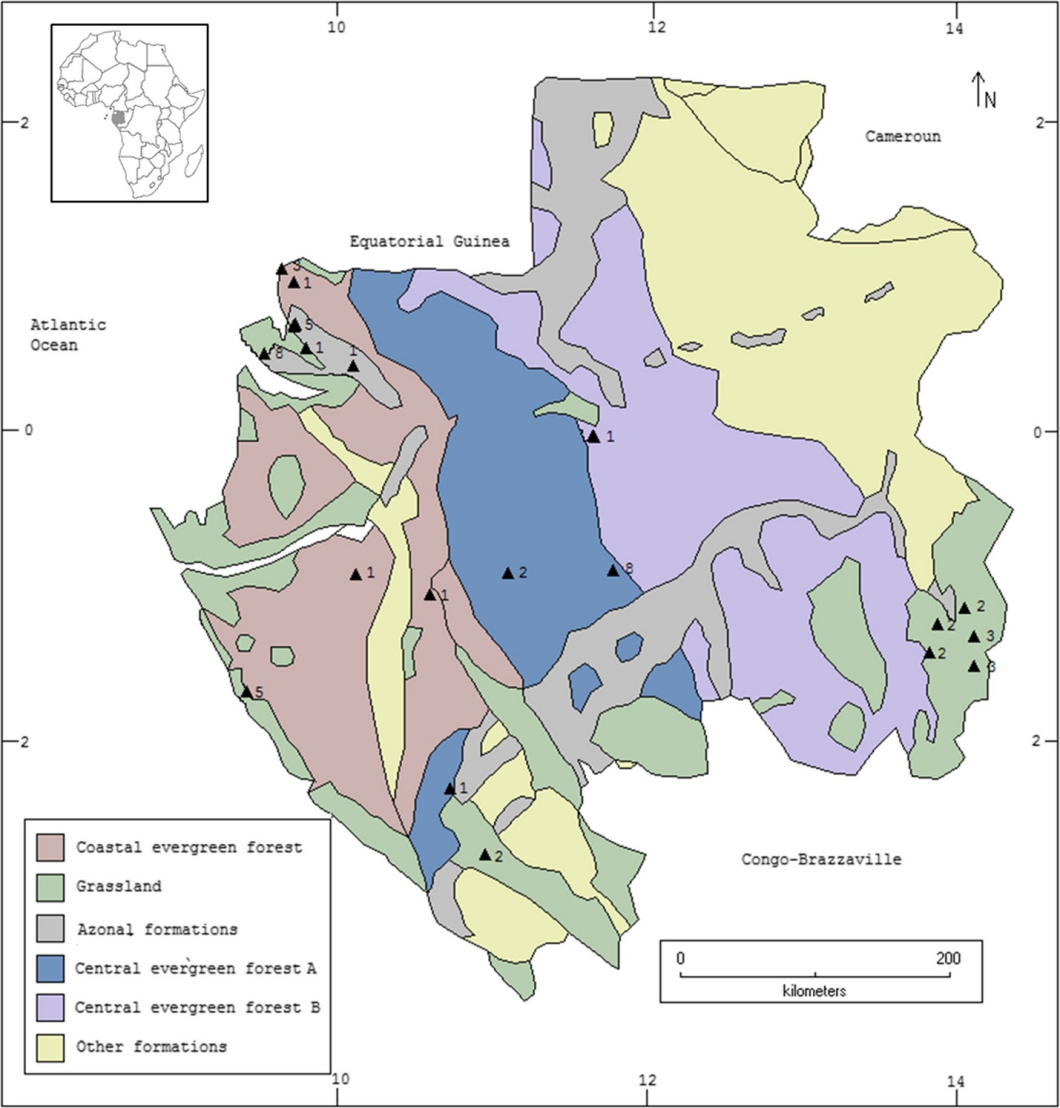
**Map of Gabon displaying the different vegetation zones.** Adapted from Caballe [27]. Triangles indicate surveyed locations. Numbers indicate informants per location.

Fieldwork was carried out in central and southern Benin (Figure 1) and in different sites throughout Gabon (Figure 2). A detailed description of the vegetation zones indicated in these figures is provided, for Benin by Adomou [22], and for Gabon by Caballe [28]. We focused on healers and adepts of traditional faiths who collected plants themselves. We only considered healers whose method of diagnose was by divination or spirit consultation. Both healers and adepts engage in plant use either by administering or receiving herbal treatments and participating in ceremonies that involve the use of plants. Sampling was done by chain referral [29], a method we chose for two reasons: 1. the lack of an accessible frame (e.g. a directory of healers and adepts), which did not allow us to draw a random sample, and 2. the expected difficulty in obtaining information from two target groups who are known to cautiously guard their trade secrets [30]. Vodoun or Orisha (in Benin) and Bwiti (in Gabon) are the collective names for several traditional religions. In Benin, these are based on the belief in the Vodoun or Orisha pantheon, i.e. gods that help the creator (Mawu-Lisa) govern the natural world, such as Sakpata, the god of earth and smallpox and Âge, the god of agriculture and forests [31]. In Gabon, Bwiti can be understood as a "religious and social institution", comprised of secret societies (e.g. Mwiri and Nyembé, two different secret societies), each with its own passage rites and ceremonies [32]. In Benin, our sample consisted of 50 informants (39 males and 11 females, 35 healers and 15 adepts) representing 9 linguistic groups and 19 traditional faiths. In Gabon, we interviewed 52 informants (30 males and 22 females, 38 healers and 14 adepts), covering 16 linguistic groups and 14 traditional faiths. Power analysis of sample size was estimated with a 90% interval using nQuery Advisor version 7.0. A value of p < 0.05 was considered statistically significant.

We conducted face-to-face, semi-structured questionnaires and asked our informants to indicate ritual plant species. For each species, we documented whether its harvest or use was associated with a taboo or merited a sacrifice or payment in kind. We only considered plant species which prohibition was related to ritual mandates and excluded plants with restrictions related to kinship or illnesses. We also asked our informants to list plants that they perceived as scarce. We then accompanied them to the places where they collected the plants mentioned during the interview in order to make herbarium specimens. Likewise, we documented plants that were actively protected by local people, that is, plants that had been removed from the wild and were cultivated by our informants in home gardens or sacred forests, as well as plants which growth was promoted in the wild. Domesticated species (e.g. maize and cassava) with a restriction status were not considered as actively protected. Plants were collected following standard botanical methods. Vouchers were deposited and identified at the national herbaria of the countries where they were collected (BEN and LBV) and the Wageningen branch of the National Herbarium of the Netherlands (WAG), now part of Naturalis Biodiversity Center (L). Scientific plant names were checked against their latest nomenclature status following The Plant List [33]. Data on habitat and distribution ranges for wild species were obtained from the PROTA database [34] and the Flore du Gabon [35, 36] and Flore analytique du Bénin [37]. Using IBM SPSS 20, we performed a Chi-square analysis to test whether: 1. the proportion of species perceived as scarce was higher among restricted plants than among non-restricted ones, 2. the proportion of officially threatened species was higher among restricted plants than among non-restricted ones, and 3. the proportion of actively protected species was higher among restricted plants than among non-restricted ones.

Following the guidelines of African Union's Model Legislation for the Protection of Indigenous Knowledge [38], informants read and signed a free and prior informed consent. Whenever informants were not able to read and write, they were informed of our intent and their verbal permission was obtained. Informants were compensated for their time with a sum of money that was convened with them prior to the interview. Research and plant export permits were obtained from our partner institutions. Digital copies of research permits are available upon request. Naturalis Biodiversity Center does not have an ethics committee or an institutional review board, but has formal partnerships with all the institutions above. Our partners confirmed that no additional ethical approval or permits were required.

## Results

### Types of taboos and sacrifices

We documented 414 plant species used for ritual purposes in Benin and 256 in Gabon. Fifty-two species were used in both countries, thus in total 618 different plant species were used. In the Additional file 1, all species are listed with their scientific and vernacular names, part used, and a brief description of their uses. The two countries differed in the way in which restrictions were transmitted. In Benin, their spiritual father or mother, that is, the person who guides the ceremony in which an adept is initiated, gives these to adepts. In the case of Fâ oracle priests, it is the interpretation of the oracle signs that are drawn during the initiation that determine what plants (and animals) are to be avoided by initiates (including the type of restriction and the plant part to be avoided). In Gabon, on the other hand, restrictions may come in manner of a vision experienced by adepts under the hallucinogenic effects of the iboga plant (*Tabernanthe iboga*) during initiation ceremonies.

We found contrasting differences in the degree of severity of restrictions related to ritual plant use in the two countries (Table 1). In Benin, the harvest of certain plant species involved intricate procedures. For example, the harvest of the cat’s-tail grass (*Sporobolus pyramidalis*) was limited to plants found at a crossroad and required the sacrifice of a ram. In Gabon, restrictions were notably *milder* and mostly limited the physical contact with plant species. Remarkably, in Gabon, collecting the fruits of the pink magic (*Massularia acuminata*) and the ofumu (*Maranthes chrysophylla*) was a taboo for any adept whose parents were still alive. We did not encounter this type of restriction related to living parents in Benin. Individual food taboos were present in both countries, especially with regard to cultivated crops. In Gabon, the leaves of taro (*Colocasia esculenta*) and manioc (*Manihot esculenta*), and pineapple fruits (*Ananas comosus*) were forbidden food items for Bwiti adepts. In Benin, cowpea (*Vigna unguiculata*), sorghum (*Sorghum bicolor*), the fruits of the African custard-apple (*Annona senegalensis*), the horned melon (*Cucumis metuliferus*), and the African oil palm (*Elaeis guineensis*) were food taboos for Fa oracle initiates whose initiation signs were governed by these species. Furthermore, in Benin some adepts mentioned not being allowed to specify the type of restriction, although they did provide the name of the plant species in question. These restrictions are included in Table 1 under *unspecified*. Moreover, restrictions that were only mentioned once were classified as *other* and include: cultivation (of maize, *Zea mays*), harvest, urination (on species such as *Mallotus oppositifolius*) and whipping (with the twigs of *Grewia carpinifolia*).

**Table 1:** **Different types of restrictions for ritual plant use in the two African countries, ranked according to frequency of citations and number of species involved**

	Benin		Gabon	
Type of restriction	Frequency	Nr of species	Frequency	Nr of species
Felling/cutting	19	12	13	3
Burning wood	13	8	0	0
Not to be used as medicine	13	8	0	0
Unspecified	13	8	0	0
Offer money/wine	9	6	22	5
Food taboo	8	5	30	7
Sacrifice livestock	8	5	0	0
Forbidden in herbal bath	6	4	0	0
Sacrifice wildlife	5	3	0	0
Touching	0	0	22	5
Collect fruits forbidden	0	0	9	2
Other	6	4	4	1

### Benin

We documented 414 plant species used in the context of religious traditions in Benin, of which 63 (15%) were restricted and 351 (85%) were not. Restricted plants were two and a half times as often officially threatened (19%) than non-restricted plants (7%) (*χ*^2^ = 10.03, df = 1, P < 0.002). Remarkably, variations between restricted plant species did not reflect affiliation to a specific traditional faith but seemed to be related more to the geographical location of our informants. For instance, adepts of Heviosso or Shango (the god of thunder) living in the Central part of the country had prohibitions on species such as the heart-fruit (*Hymenocardia acida*) and the groundnut tree (*Ricinodenron heudelottii*). In contrast, their counterparts of the same traditional faith in the South of Benin mentioned restrictions on species such as the African border tree (*Newbouldia laevis*), the Senegal prickly-ash (*Zanthoxylum zanthoxyloides*), and the yellow mombin (*Spondias mombin*). Incidentally, these restrictions also applied to adepts of other faiths in south Benin. The kapok (*Ceiba pentandra*) and the iroko tree (*Milicia excels*a), however, were restricted species shared by all groups and in all regions. Table 2 shows the most salient restricted species in Benin.

**Table 2:** **Restricted and threatened plants in Benin, ranked according to their citation frequency**

Restricted species	Citation frequency (% of informants)	Official threat status	Perceived scarcity (% of informants)	Habitat	Habit	Distribution
Kapok tree (*Ceiba pentandra*)	20		23	Primary forest to settlements	Large tree	Pantropical
African border tree (*Newbouldia laevis*)	18		0	Secondary forest to settlements	Small tree	Pantropical
Senegal prickly-ash (*Zanthoxylum zanthoxyloides*)	16	VU^b^	46	Savanna and dry forest	Small tree	West Africa
Iroko tree^a^ (*Milicia excelsa*)	14	EN^b^/ VU^c^	15	Primary to secondary deciduous forest	Large tree	West, Central, and East Africa
Heart-fruit (*Hymenocardia acida*)	12		0	Savanna to deciduous woodland	Shrub	Tropical Africa
Cowpea (*Vigna unguiculata*)	12		0	Cultivated fields, settlements	Vine	Pantropical
Yellow mombin (*Spondias mombin*)	10		0	Secondary deciduous forest	Medium-sized tree	Pantropical
Groundnut tree (*Ricinodendron heudelotii*)	10		0	Secondary humid forest to fallows	Medium-sized tree	West, Central and East Africa
Nicker tree (*Caesalpinia bonduc*)	0	EW^b^	53	Disturbed sites	Shrub	Pantropical
African locust tree (*Parkia biglobosa*)	0		46	Savanna to cultivated fields	Small tree	West and Central Africa
Wild banana (*Ensete livingstonianum*)	0	CR^b^	30	Savanna	Large herb	West Africa
Yellow plum (*Ximenia americana*)	0		30	Savanna to dry forests	Small tree	Pantropical

^a^Important timber species [39]. ^b^Locally threatened [40]. ^c^Globally threatened [41]. EN = Engangered, VU = Vulnerable, CR = Critically endangered, EW = Extinct in the wild.

Perceived scarcity, on the other hand, showed little variation between informants in southern and central Benin. Restricted plants were twice as often perceived as scarce (29%) than non-restricted ones (12%) (*χ*^2^ = 11.88, df = 1, P < 0.001) (Table 2 and Additional file 1). Plants such as the yellow plum (*Ximenia americana*), the wild banana (*Ensete livingstonianum*), and the poison arrow wood (*Strophanthus hispidus*), however, showed a perceived scarcity limited to a single location in central Benin. People most often attributed the disappearance of plant species to urban and agricultural development (92% of informants), but also to the export-oriented exploitation of timber species (8%).

### Gabon

We recorded 256 plant species used for religious traditions, of which 23 (9%) were restricted species and 233 (91%) were not. Compared to our results in Benin, restrictions in Gabon appeared to follow neither a geographical nor a faith-related pattern. Rather, they included large forest trees and a few cultivated crops. Restricted plant species were more often perceived as scarce (12%) than non-restricted ones (7%) (*χ*^2^ = 19.47, df = 1, P < 0.001). The majority of informants (87%) identified timber exploitation as the main cause for the scarcity of plants. Accordingly, the most frequently mentioned plants in this category corresponded to important species in the international timber trade (Table 3 and Additional file 1). Fewer informants (13%) attributed rareness to the seasonal availability of plants such as herbs that were difficult to find during the dry season. Finally, restricted plant species were slightly more often officially threatened (9%) than non-restricted plants (6%), but this difference was not significant.

**Table 3:** **Restricted and threatened plants in Gabon, ranked according to their citation frequency**

Restricted species	Citation frequency (% of informants)	Official threat status	Perceived scarcity (% of informants)	Habitat	Habit	Distribution
Kevazingo ^a^(*Guibourtia tessmannii*)	33		42	Primary evergreen forest	Large tree	Cameroon, Equatorial Guinea, Gabon
Moabi ^a^(*Baillonella toxisperma*)	26	VU^b^	28	Primary rain forest	Large tree	Nigeria to Gabon (and DR Congo)
Red anzem (*Copaifera religiosa*)	26		23	Primary lowland rain forest	Large tree	Cameroon to DR Congo
Kapok tree (*Ceiba pentandra*)	20		15	Primary forest to settlements	Large tree	Pantropical
Pink magic (*Massularia acuminata*)	20		15	Primary forest to settlements	Shrub	West and Central Africa
Ofumu (*Maranthes chrysophylla*)	13		0	Primary to secondary forest	Large tree	West and Central Africa
Spider tresses (*Strophanthus gratus*)	13		7	Primary moist forest	Liana	West and Central Africa
Horseradish tree (*Drypetes gossweileri*)	6		7	Primary to secondary semi-deciduous forest	Medium-sized tree	Central Africa
Bitter kola (*Garcinia kola*)	0	VU^b^	15	Primary to secondary evergreen forest	Medium-sized tree	West and Central Africa
Ordeal tree (*Erythrophleum ivorense*)	0		15	Primary to secondary evergreen forest	Large tree	West and Central Africa
African padauk ^a^(*Pterocarpus soyauxii*)	0		15	Primary to secondary evergreen forest	Large tree	Nigeria to Angola

^a^Important timber species [42, 43]. ^b^Globally threatened [41]. VU = Vulnerable.

### Emic explanations for taboos and sacrifices

When asked, our informants’ most common explanation to the existence of taboos and sacrifices in Benin (34% of interviewees) was that these were created in order to keep the union, peace, or happiness of the individual, the family, and the community. In Gabon, respondents most often indicated (60%) that restrictions were practices that had permitted ancestors to exist in equilibrium with their entourage, and as such, they should be kept in order to assure the survival of present generations. In Benin, that response was only second in importance (27%). Another common interpretation was that restrictions were a means to avoid disease (23% of informants in Benin and 32% in Gabon), followed by the belief that restrictions existed because "it is the spirits’ will [that there are taboos]" (11% in Benin). Only a few informants (6% in Benin and 8% in Gabon) indicated not knowing why restrictions existed.

We also asked our respondents to explain what the consequences of not respecting such restrictions would be. The most prevalent response was "illness and eventual death" (42% in Benin, and 69% in Gabon). In Benin, another common response was that breaking taboos would bring misfortune to the entire family (25%). In both countries, informants indicated madness to be a punishment for sinners (7% in Benin and 21% in Gabon). The loss of spiritual power, the detriment of the tradition, and the dissolution of the family were also regarded as possible consequences of disobeying restrictions. Last, in response to our question "what can one do in order to be forgiven for breaking a taboo?" all respondents agreed that ritual ceremonies were necessary in order to be forgiven by the spirits or ancestors (including the sacrifice of livestock, usually a chicken or a goat).

### Active protection of restricted vs. non-restricted plant species

We encountered different ways in which people actively protected wild plant species used for ritual purposes. In Benin, it was common to find full grown trees of species such as the baobab (*Adansonia digitata*), the iroko (*Milicia excelsa*), and the kapok (*Ceiba pentandra*) in forests patches (or even roadsides) wrapped with a white cloth tied at breast height. The cloth, usually covered with a stain of oil palm mixed with maize flour (which are common ingredients in rituals), was an indication that the tree was a sacred one (i.e. a dwelling place for spirits) and therefore should be spared from felling. Often, adepts and healers would carefully uproot plants from their wild surroundings to tend them at home gardens or sacred forests. Also plants’ growth would be promoted in the wild by weeding around them or by pruning neighboring trees to secure that these plants would have better access to water and light. When asked, informants indicated that growing plants that were perceived as scarce, such as the wild banana (*Ensete livingstonianum*) and the poison arrow wood (*Strophanthus hispidus*) at home was the only way to secure their survival. In Benin, roughly, 50% of all restricted species were actively protected, and we found a significant relation between these two (Table 4). In contrast, far fewer restricted plants were protected in Gabon, where only 13% of the total were taken from the wild to be planted in home gardens (notably, iboga *Tabernanthe iboga*) or spared from felling (particularly *Baillonella toxisperma* and *Copaifera religiosa*). Nonetheless, this percentage was not significantly different from that of non-restricted plants.

**Table 4:** **Restricted, non-restricted, and actively protected plants compared**

		Actively protected Nr. of species (% of total)				
	Restriction	Yes	No	Total	χ ^2^	P
Benin	Yes	22 (5)	41 (10)	63	7.255	0.007
	No	69 (17)	282 (68)	351		
Gabon	Yes	3 (1)	20 (8)	23	0.400	0.527
	No	21 (10)	212 (91)	233		

## Discussion

Our results showed that restricted plant species are significantly more often officially threatened, perceived as scarce, and actively protected by adepts and healers in Benin than non-restricted plants. These findings support earlier studies that suggested taboos as social institutions for resource conservation [44–47]. At the same time, however, our findings contend with previous evidence that taboos occur only amongst populations with high availability and choice of resources [12, 25]. We encountered three times as many restricted plants in Benin, the least species-rich, least forested, and most densely populated of the two countries of study. On the other hand, food taboos were far more common in Gabon than in Benin. These results are consistent with the "taboo-as-a-luxury" premise in the case of food taboos. Therefore, this combination of findings calls for further scrutiny of Hardesty’s niche concept in human ecology, especially with respect to the insufficiency of its theoretical framework [48].

Where there is no indication of a link between restricted species and officially threatened ones, and actively protected plants in Gabon, restricted and perceivedly scarce plants did show a significant relation. This finding becomes particularly relevant when looking at species such as kevazingo (*Guibourtia tessmannii*) and the red anzem (*C. religiosa*). Both were perceived as scarce by our informants, have a narrow distribution [34], and are exploited for timber, but their threat status has not yet been officially evaluated [41]. Lykke [49] considered that consensus in response to structured questions among people with different backgrounds is a good indication that a true process is described. In our study, her claim is exemplified by the types of restrictions found in Benin that reflected a geographical pattern (and not a faith-based one), and in both countries by the congruency between the restrictions associated with plants and their perceived scarcity. Considering these two trends in our results, we can state that local people could be a potentially reliable source of information on endangered species. Moreover, these results encourage us to support the emphasis other authors have made on the little attention plants receive when it comes to the Red List [50].

Finally, one might ask to what extent do restrictions such as taboos and ritual sacrifices contribute to the conservation of plant species. While we found that there is a relation between restricted and actively protected species in Benin, we lack the evidence to ascertain that restrictions will ultimately result in the sustainable use of ritual plants. Taboos or sacrifices do not seem to apply to commercial loggers. In spite of being one of the most sacred trees in Benin, the iroko (*Milicia excelsa*) is still largely exploited as timber product [51]. The same applies to the ritually important okume (*Aucoumea klaineana*) in Gabon. The belief in the ritual power of plants, apart from posing a potential, can also be an obstacle to their conservation. Because of the supernatural powers that are often attributed to them, the harvesting of such plants for commercial purposes is a profitable activity. Hundreds of ritual plants are traded on medicinal plant markets in West Africa [52–54] and Gabon [55]. Although restricted plants are often protected, their products are still sold and purchased by adepts. As we have no evidence of the intent of all actors [12, 56], we do not know the ultimate effect of protection and commercial harvesting for ritual purposes on these plants’ survival. Therefore, studies on ritual plant use should focus not only on potentials but also on constraints for conservation.

## Conclusions

This study set out with the aim of providing evidence-based data about the role of religious traditions in nature conservation. We are certain that it will be especially valuable in informing conservation planning by proposing a simple methodological approach to obtain baseline data on endangered plant species. In this study, we were able to confirm the relationship between restrictions on plant use and the intent to conserve these plant species. It also substantiated the premise that ritual restrictions reflect plant scarcity. The large number of restricted plants and the intentions of local people to cultivate or to protect ritual species in Benin suggests a form of adaptive management where restrictions related to ritual plant use are more prevalent in degraded landscapes. In species-rich and heavily forested Gabon, lower numbers of restricted species and fewer signs of the active protection of plants by informants reflected a greater availability of plants. Still, restricted species were also more often perceived as scarce, especially primary rainforest trees subject to commercial timber exploitation. Finally, our results contribute to the discussion on the purpose of taboos by demonstrating that in the case of non-food taboos, restrictions on plant use do not necessarily occur among people with high availability of resources.

## Electronic supplementary material

Additional file 1: **Complete list of ritual plants in Benin and Gabon including restriction, threat, active protection, and domestication status, as well as perceived scarcity.** (XLS 185 KB)

## Abbreviations

BEN: National Herbarium of Benin
LBV: National Herbarium of Gabon
WAG: National Herbarium of the Netherlands Wageningen Branch
L: National Herbarium of the Netherlands
IUCN: International Union for the Conservation of Nature
PROTA: Plant Resources of Tropical Africa.
